# Atypical Multisegmental Involvement in Suspected Ischemic Colitis: A Case Report

**DOI:** 10.7759/cureus.117150

**Published:** 2026-09-29

**Authors:** Henry K Mah, John A Spallanzani, Mike De Leonardis, Alex Shu, Roxana Lazarescu

**Affiliations:** 1 Internal Medicine, Wyckoff Heights Medical Center, New York, USA

**Keywords:** conservative medical management, elderly patient management, gastrointestinal (gi) bleeds, ischemic colitis, multi-segmental colonic involvement

## Abstract

We report a case compatible with ischemic colitis in a 68-year-old man with hypertension, coronary artery disease treated with three stents, peripheral arterial disease, and diabetes mellitus who presented with acute abdominal pain, vomiting, and bloody diarrhea. Initial white blood cell count was 22.2 K/µL, and serum lactate was 0.8 mmol/L. Contrast-enhanced CT demonstrated multisegmental colitis. Colonoscopy showed severe, noncontiguous inflammation involving the rectum, sigmoid colon, hepatic flexure, ascending colon, and cecum, with a normal terminal ileum. Sharply demarcated ulceration and dusky mucosa favored ischemia, although these findings were not diagnostic. The patient remained hemodynamically stable without peritoneal signs and improved with bowel rest, intravenous fluids, ceftriaxone plus metronidazole, surgical consultation, and serial abdominal examinations. No operation was performed. The ischemic mechanism remained indeterminate: dedicated vascular imaging and colonic histopathology were unavailable, stool studies were not obtained, and infectious or other mimics could not be excluded definitively. This case illustrates that suspected ischemic colitis can have atypical rectal and right-sided involvement despite a relatively benign examination and a lactate within the reference range. In this clinically stable patient, close surgical observation supported continued nonoperative treatment despite guideline features of severe disease.

## Introduction

Ischemic colitis is the most common form of gastrointestinal ischemia and usually affects older adults with vascular disease. It commonly presents with abrupt abdominal pain followed by hematochezia [1,2]. Most cases result from a transient reduction in colonic blood flow rather than a fixed arterial obstruction, particularly in patients with hypertension, diabetes mellitus, coronary artery disease, and peripheral arterial disease [2,3]. Typical injury occurs in watershed regions such as the splenic flexure and rectosigmoid junction [1].

Atypical distribution can complicate diagnosis. Right-sided disease is associated with worse outcomes, while rectal ischemia is uncommon because the rectum receives collateral blood flow from both mesenteric and iliac arterial systems [4-6]. Rectal or noncontiguous multisegmental inflammation also broadens the differential diagnosis to include infectious colitis, inflammatory bowel disease, medication-related injury, vasculitis, and stercoral injury. Acute mesenteric ischemia must also be considered when pain is severe, right-sided disease predominates, or the clinical course deteriorates. A normal serum lactate does not exclude early intestinal ischemia [7].

We describe a case compatible with ischemic colitis in a hemodynamically stable older man with substantial vascular comorbidity and severe, noncontiguous inflammation involving the rectum and multiple right- and left-sided colonic segments. The case illustrates the diagnostic limits of a reassuring examination and lactate value and the importance of serial reassessment when nonoperative treatment is pursued. Neither a non-occlusive mechanism nor ischemia itself was definitively established.

## Case presentation

A 68-year-old man with hypertension, coronary artery disease treated with three stents, peripheral arterial disease, diabetes mellitus, and a prior rib fracture presented to the emergency department after acute generalized abdominal pain, weakness, vomiting, and bloody diarrhea began the previous night. The pain increased from 7/10 to 8/10, had a grinding quality, and was most prominent in the lower abdomen with radiation to the right side. He had been unable to tolerate oral intake since symptom onset.

He denied recent travel, sick contacts, trauma, raw or undercooked food exposure, fever, chills, diaphoresis, constipation, abdominal distension, anorexia, hematuria, tobacco use, alcohol use, and illicit drug use. A complete medication reconciliation was unavailable, including use of antiplatelet agents, anticoagulants, nonsteroidal anti-inflammatory drugs, vasoconstrictors, or other medications relevant to bleeding or colonic injury. No hypotensive or other low-flow event was documented before presentation; an undocumented episode before arrival cannot be excluded.

On arrival, temperature was 99.5°F, blood pressure was 135/85 mmHg (mean arterial pressure was 101 mmHg), heart rate was 95 beats/min, respiratory rate was 16 breaths/min, and oxygen saturation was 97% on room air. Triage pain was rated 9/10. He was alert, well-appearing, and in no acute distress. The abdomen was soft with hyperactive bowel sounds and mild epigastric and lower abdominal tenderness, without guarding, rebound, distension, or other peritoneal signs. Fecal occult blood testing was positive. Other examined systems were unremarkable. Numerical vital signs after fluid administration were not available, and the initial readings do not establish whether a transient low-flow event occurred before arrival.

The initial working diagnosis was gastroenteritis, with diverticulitis also considered. Marked neutrophilic leukocytosis prompted empiric intravenous ceftriaxone and metronidazole, while infectious colitis remained in the differential. The exact antibiotic start date, duration, and stop date were unavailable. The rationale for broad-spectrum antibiotics in suspected moderate or severe colon ischemia is discussed below; improvement in this case cannot be attributed specifically to this regimen.

On hospital day 1, contrast-enhanced CT of the abdomen and pelvis demonstrated marked mural edema involving the cecum and right colon and the hepatic flexure through the mid-transverse colon, with less advanced edema from the mid-to-lower left colon through the proximal sigmoid colon (Figure 1). The available report did not describe pneumatosis, portal venous gas, free intraperitoneal air, ascites, a mesenteric vascular occlusion, or small-bowel ischemia. This routine examination was not documented as multiphasic CT angiography, so the absence of a reported occlusion does not establish mesenteric vessel patency or exclude an occlusive process.

**Figure 1:**
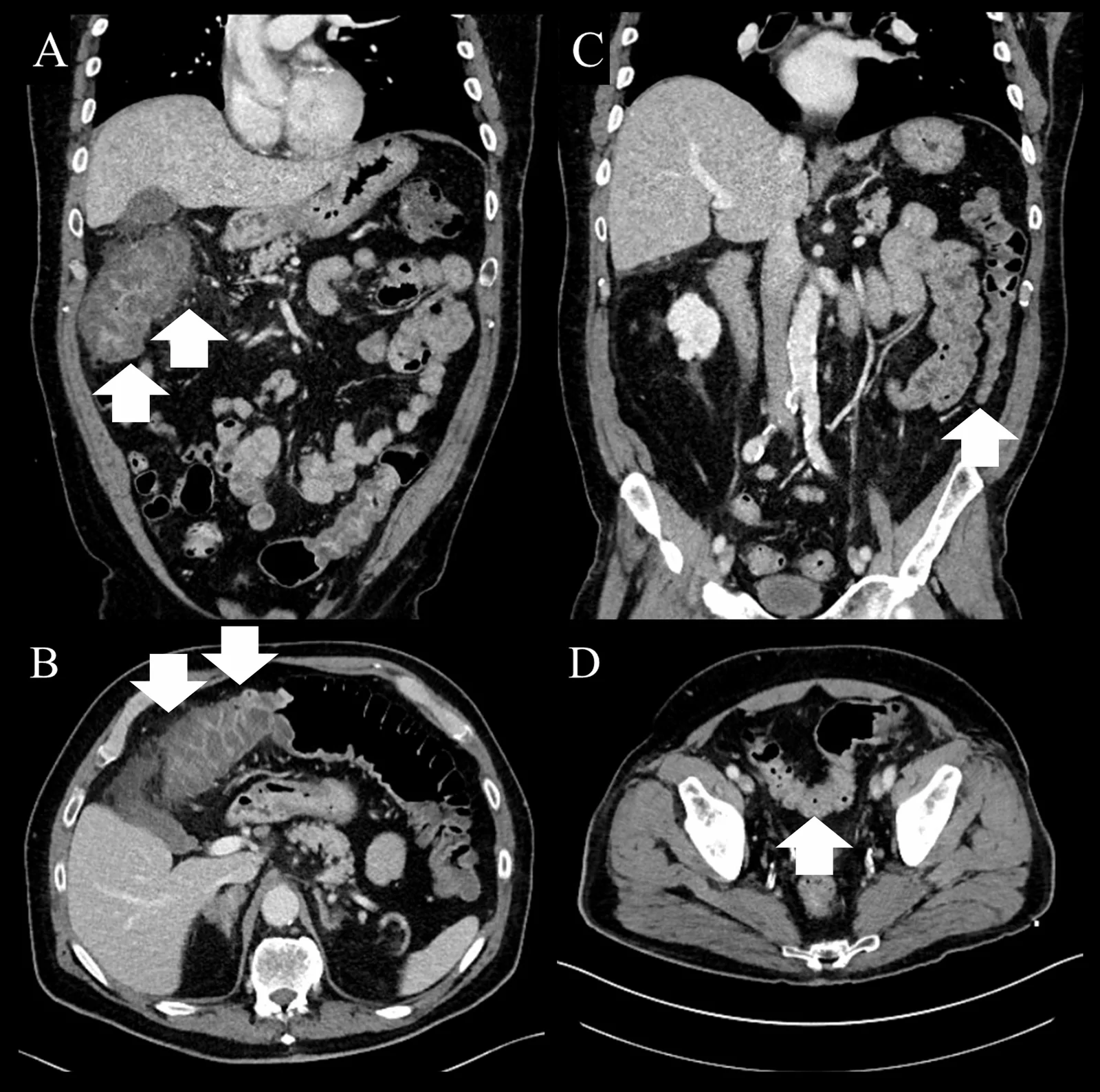
Contrast-enhanced CT of the abdomen and pelvis Arrows indicate marked mural edema in the cecum and right colon (A) and hepatic flexure through the mid-transverse colon (B), with less advanced edema from the mid-to-lower left colon (C) through the proximal sigmoid colon (D). The available report did not describe pneumatosis, portal venous gas, free air, or small-bowel ischemia. Dedicated multiphasic CT angiography was not performed, so mesenteric arterial occlusion was not definitively excluded.

Early in the hospitalization, esophagogastroduodenoscopy was performed because an upper gastrointestinal source of bleeding remained possible. It showed a regular Z-line 38 cm from the incisors, erythematous gastritis in the antrum and fundus, and acute duodenitis, without active or recent upper gastrointestinal bleeding (Figure 2). Gastric biopsies were obtained, but the pathology report, including *Helicobacter pylori* status and assessment for medication-related injury, was not available. The upper endoscopic findings therefore did not explain the patient's hematochezia.

**Figure 2:**
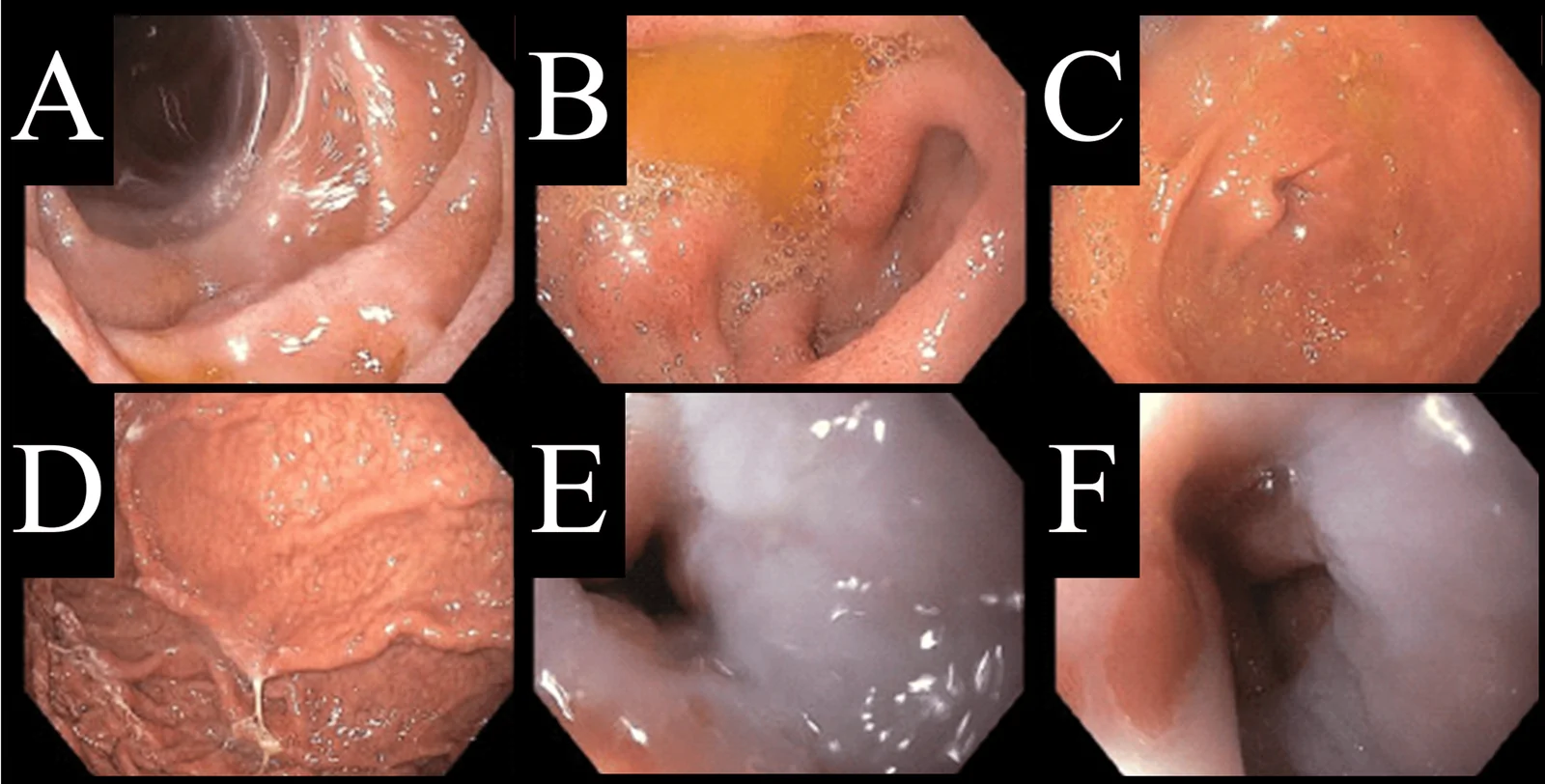
Upper gastrointestinal endoscopy The third portion of the duodenum (A) and second portion of the duodenum (B) showed duodenitis; the gastric antrum (C) and body (D) showed erythematous gastritis. The lower third of the esophagus is shown in panels E and F. No active or recent upper gastrointestinal bleeding was identified. Gastric biopsy results were not available.

Subsequent colonoscopy showed severe, noncontiguous inflammation involving the rectum, sigmoid colon, hepatic flexure, ascending colon, and cecum (Figure 3). The rectum showed patchy erythema and edema. The sigmoid and right-sided segments showed friable, edematous mucosa with segmental ulceration, adherent exudate, areas of dusky discoloration, and a sharply demarcated transition between affected and less affected mucosa. These appearances favored ischemic injury but are not specific to it. The terminal ileum was normal. No gangrene was documented endoscopically, and there were no procedural complications. The available report did not separately establish involvement or sparing of every intervening transverse or descending segment; the distribution is therefore described as multisegmental rather than pancolonic. Colonic histopathology was unavailable, an important limitation given the rectal and discontinuous involvement.

**Figure 3:**
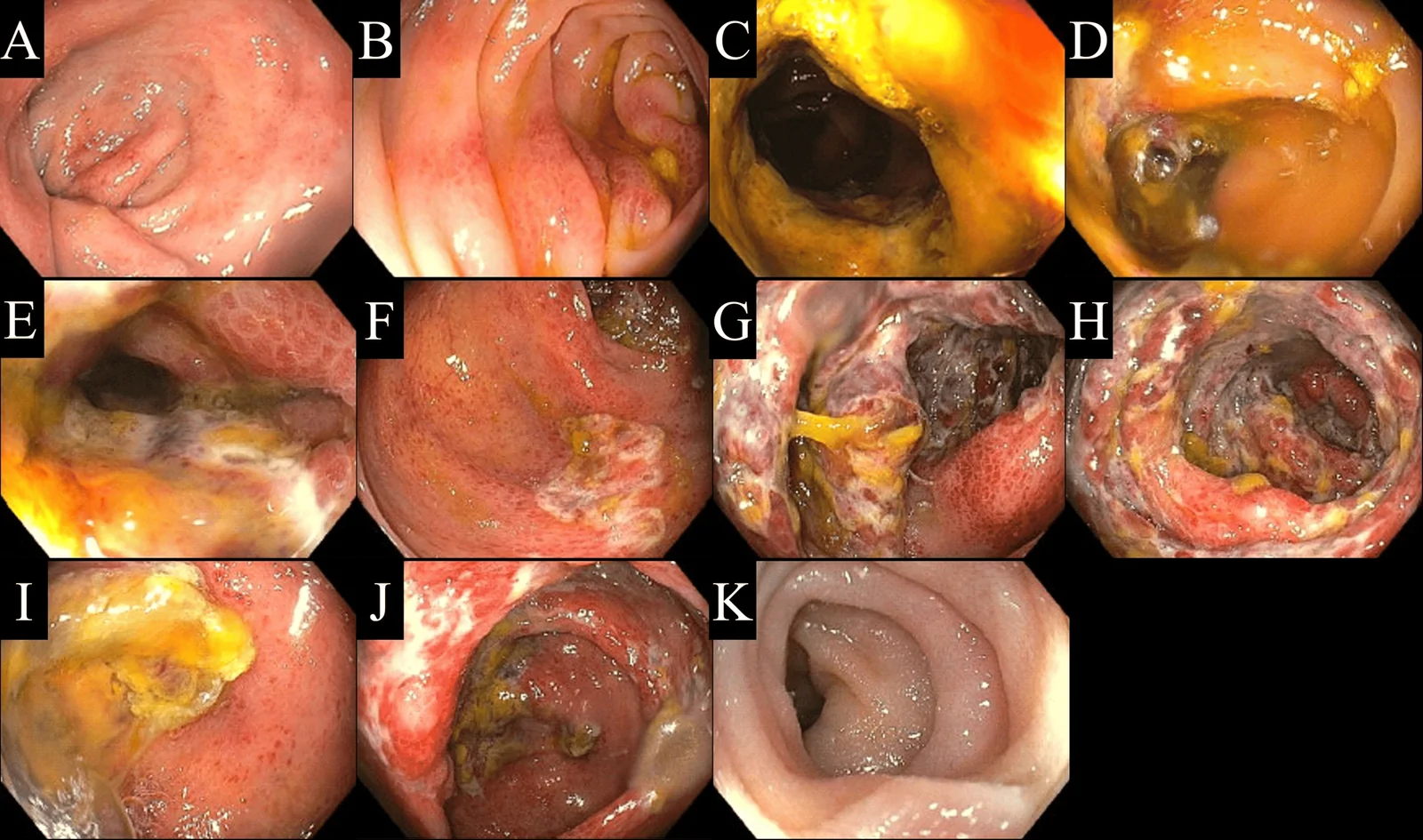
Colonoscopy The rectum (A) shows patchy erythema and edema. The sigmoid colon (B-E) shows a sharply demarcated progression from erythema and edema to extensive ulceration, adherent exudate, and dusky-appearing mucosa. The hepatic flexure (F) shows focal erythema and ulceration. The ascending colon (G-H), appendiceal orifice (I), and ileocecal valve (J) show severe circumferential ulceration, friability, edema, and exudate. The terminal ileum (K) appears normal. The sharply demarcated transition, segmental ulceration, friability, and dusky mucosa favored ischemic injury but were not specific for it. Gangrene was not documented, and colonic histopathology was unavailable.

General surgery was consulted because of the extensive and right-sided disease. In the absence of peritoneal signs, persistent hemodynamic instability, documented gangrene, perforation, pneumatosis, or clinical deterioration, the team recommended bowel rest, nil per os status, intravenous fluids, antimicrobial therapy, and serial abdominal examinations. Repeat colonoscopy in two months was recommended to assess mucosal healing; follow-up endoscopic results were not available for this report.

Selected laboratory trends are shown in Tables 1, 2. Serum lactate was 0.8 mmol/L, and lactate dehydrogenase was 158 U/L; both were within their reported laboratory reference ranges. INR (International Normalized Ratio) was 1.21, and activated partial thromboplastin time was 28.1 seconds. C-reactive protein, venous or arterial pH, and blood culture results were unavailable. Stool cultures, *Clostridioides difficile* testing, and gastrointestinal pathogen testing were not obtained. The calculated anion gap remained normal.

**Table 1:** Selected biochemical profile during hospitalization Day 2 values were not available. The anion gap was calculated as sodium − (chloride + bicarbonate), without potassium. Serum lactate was 0.8 mmol/L, and lactate dehydrogenase was 158 U/L; both were within their reported reference ranges.

Test	Day 1	Day 3	Day 4	Day 5	Day 6	Reference range
Sodium, mmol/L	133	133	132	136	136	136-145
Chloride, mmol/L	99	98	98	102	103	98-107
Bicarbonate (serum CO_2_), mmol/L	26	25	26	29	27	21-32
Calculated anion gap, mmol/L	8	10	8	5	6	Varies by laboratory
Blood urea nitrogen, mg/dL	16	10	8	8	7	7-18
Creatinine, mg/dL	1.25	0.95	1.14	1.09	1.04	0.55-1.30
Albumin, g/dL	3.8	2.8	2.8	2.2	2.5	3.4-5.0
Glucose, mg/dL	218	195	166	128	148	74-106

**Table 2:** Selected hematologic profile during hospitalization NA indicates not available or not collected. No transfusion was documented in the available source material. The hemoglobin decline occurred during ongoing hematochezia and intravenous fluid therapy; the relative contributions of blood loss and hemodilution could not be determined.

Test	Day 1	Day 3	Day 4	Day 5	Day 6	Reference range
White blood cell count, K/microL	22.2	25.7	23.6	14.6	12.2	4.5-11.0
Hemoglobin, g/dL	15.4	14.0	13.9	11.8	12.4	13-17
Hematocrit, %	45.4	NA	41.0	36.2	37.1	41-51
Platelets, K/microL	192	142	141	149	191	130-400
Neutrophils, %	89.4	NA	84.9	78.4	71.1	50-70

The patient had one episode of bloody diarrhea daily from admission through hospital day 4, no bowel movement on day 5, a normal nonbloody bowel movement on day 6, and no bowel movement on day 7. His abdomen was tender in the epigastric and lower regions on day 1, nontender on days 2-4, mildly tender in the lower abdomen on day 5, and nontender with minor right lower quadrant discomfort on day 6. By day 7, the abdomen was nontender. Throughout the hospitalization, his abdomen remained soft and nondistended with active bowel sounds and no peritoneal signs. Leukocytosis declined from 25.7 K/microL on day 3 to 12.2 K/microL on day 6. Hemoglobin decreased from 15.4 g/dL on day 1 to a nadir of 11.8 g/dL on day 5 and rose to 12.4 g/dL on day 6. No hemodynamic instability or transfusion was documented.

Repeat colonoscopy approximately two months later was recommended to assess mucosal healing. No outpatient symptom assessment, interval laboratory testing, subsequent vascular evaluation, readmission record, or follow-up colonoscopy result was available for this report; sustained recovery after discharge cannot be assessed.

## Discussion

The diagnosis in this case was clinical and endoscopic rather than histologically confirmed. Sudden abdominal pain and hematochezia in a patient with coronary and peripheral arterial disease, CT evidence of segmental colitis, sharply demarcated transitions, dusky mucosa, and segmental ulceration at colonoscopy supported ischemic colitis. Evaluation of suspected bowel ischemia integrates clinical findings, imaging, and endoscopic assessment [8]. The rapid improvement with supportive treatment was compatible with this interpretation but was not diagnostic. Colonic biopsy results were unavailable, and stool infectious studies were not obtained. These gaps matter particularly because rectal and discontinuous inflammation can also occur with infectious proctocolitis, inflammatory bowel disease, medication-related injury, vasculitis, or stercoral injury. A complete medication history was unavailable, limiting assessment of both bleeding risk and potential causes of colonic injury. Accordingly, the case is best described as compatible with ischemic colitis rather than as a confirmed example of it.

Ischemic colitis often affects older adults with cardiovascular comorbidity and typically involves watershed regions. Rectal involvement is uncommon because of collateral blood supply from the mesenteric and iliac circulations [4]. Severe injury, gangrene, perforation, and need for surgery are associated with worse outcomes [9-13]. Isolated right-colon ischemia has been associated with worse outcomes than left-sided disease [5,6]. This patient had right-sided involvement together with left-sided and rectal abnormalities; the disease was neither isolated to the right colon nor documented as pancolonic. Its extensive and atypical distribution increased concern for serious ischemia and for competing diagnoses.

The mechanism remained indeterminate. Routine contrast-enhanced CT did not report a vascular occlusion, but dedicated multiphasic CT angiography or another vascular study was unavailable, and arterial occlusion was not definitively excluded. No hypotensive or other low-flow trigger was documented, although an event before admission cannot be ruled out. The case therefore should not be labeled specifically non-occlusive. Lactate was 0.8 mmol/L, but a value within the reference range does not exclude colon ischemia or early acute mesenteric ischemia [7]. In a patient with right-sided disease and severe pain, vascular assessment deserves particular consideration; the American College of Gastroenterology (ACG) guideline recommends multiphasic CT angiography when isolated right-colon ischemia is suspected, or acute mesenteric ischemia cannot be excluded [14].

The ACG guideline grades suspected colon ischemia as severe when more than three specified poor-outcome factors are present or when certain high-risk findings occur [14]. This patient had three factors at admission: male sex, serum sodium below 136 mmol/L, and white blood cell count above 15 × 10⁹/L. Colonoscopic mucosal ulceration added a fourth, satisfying the guideline definition of severe disease. Hemoglobin subsequently fell below 12 g/dL on hospital day 5, adding another factor at that time. He had no documented hypotension at presentation, tachycardia above 100 beats/min, elevated blood urea nitrogen, or elevated lactate dehydrogenase. The severe classification warranted urgent surgical assessment, broad-spectrum antibiotics, and close monitoring; it did not, by itself, establish transmural necrosis or require immediate resection. General surgery evaluated him, and serial examinations showed no peritoneal signs, documented gangrene, perforation, pneumatosis, persistent instability, or clinical deterioration.

The ACG guideline advises considering antimicrobial treatment in moderate or severe colon ischemia, although the supporting evidence is of very low quality [14]. Ceftriaxone plus metronidazole was given empirically in this case, while infection was also considered. The patient's improvement occurred during bowel rest, intravenous fluids, antibiotic treatment, and repeated clinical assessment. A single case cannot establish that this specific antibiotic combination improved the outcome or distinguish its effect from the other interventions or natural recovery.

The nonoperative decision depended on the patient's observed course rather than a favorable lactate or a mild initial examination alone. The abdomen remained soft without peritonitis, hematochezia resolved, white blood cell count fell from a peak of 25.7 K/µL on day 3 to 12.2 K/µL on day 6, and no persistent hemodynamic instability or organ failure was documented. Ongoing reassessment was essential because right-sided and extensive disease can worsen despite initially reassuring findings. Peritonitis, gangrene, perforation, portal venous gas or pneumatosis, massive ongoing bleeding, persistent instability, or clinical deterioration would have prompted renewed urgent surgical evaluation [14].

The principal limitations are the lack of colonic histopathology, infectious stool studies, a complete medication history, dedicated mesenteric vascular imaging, and documented outpatient follow-up. Consequently, ischemia is favored but not proven, its mechanism cannot be assigned, and a competing diagnosis or later recurrence cannot be excluded. This case illustrates how colonoscopy and close surgical observation informed treatment in one patient with an atypical pattern; it does not establish a general nonoperative strategy for extensive or right-sided colitis.

## Conclusions

In this case, acute pain and hematochezia, vascular comorbidity, CT findings, and sharply demarcated, dusky segmental ulceration at colonoscopy were compatible with ischemic colitis involving right- and left-sided segments and the rectum. Serum lactate was 0.8 mmol/L, and the initial examination showed no peritoneal signs; neither observation rules out clinically important ischemia. The mechanism remained uncertain because dedicated vascular imaging was unavailable, while absent colonic histopathology and stool testing limited diagnostic certainty. Although the patient met guideline criteria for severe disease, he improved during supportive treatment with early surgical involvement and close serial reassessment. This single case supports careful individualized observation in a stable patient and cannot establish that extensive or right-sided disease is generally safe to treat without surgery.
